# Homeless Patient With Community-Acquired Methicillin-Resistant Staphylococcus aureus Brain Abscesses

**DOI:** 10.7759/cureus.39667

**Published:** 2023-05-29

**Authors:** Misbah Jilani, Mellisa Renteria, Michael J Brockman, Suvarna Guvvala

**Affiliations:** 1 Internal Medicine, Texas Tech University Health Sciences Center El Paso, El Paso, USA

**Keywords:** leave against medical advice, cerebral abscess, homeless persons, mrsa brain abscess, community-acquired methicillin-resistant staphylococcus aureus (ca-mrsa)

## Abstract

Brain abscess secondary to community-acquired methicillin-resistant *Staphylococcus aureus* (CA-MRSA) infection is a rare, yet highly fatal disease. This article presents the case of a 45-year-old homeless female with a medical history of bipolar disorder, seizure disorder, and substance use disorder who was admitted with altered mental status. Laboratory tests on admission revealed neutrophil-predominant leukocytosis, elevated inflammatory markers (erythrocyte sedimentation rate [ESR], C-reactive protein [CRP]), and lactic acid. MRI brain demonstrated multiple cerebral abscesses with surrounding edema and sagittal vein thrombosis. The patient was initiated on broad-spectrum antibiotics and underwent a right-sided minimally invasive needle biopsy of the abscess and left frontal craniotomy for abscess evacuation, the culture of which confirmed the diagnosis of MRSA infection. As the patient did not have any hospitalization or procedure in the recent past, a diagnosis of CA-MRSA was made. The patient's clinical status improved following the procedure and antibiotic administration, but she left against medical advice before completing treatment. This case highlights the importance of early recognition and aggressive management of CA-MRSA infections, especially in vulnerable populations such as the homeless.

## Introduction

Methicillin-resistant *Staphylococcus aureus *(MRSA) infection is a major public health concern due to its high prevalence and resistance to multiple antibiotics [1]. Community-acquired methicillin-resistant *Staphylococcus aureus* (CA-MRSA) infections are becoming increasingly common, with neurologic manifestations being a rare but serious complication [1]. Brain abscesses caused by MRSA are particularly challenging to diagnose and manage due to their rarity and nonspecific presentation [2]. In this case report, we describe the case of a 45-year-old homeless female with a history of bipolar disorder, seizure disorder, and substance use disorder who presented with altered mental status. An extensive workup revealed a CA-MRSA infection resulting in multiple cerebral abscesses and sagittal vein thrombosis.

The diagnosis of cerebral abscesses can be challenging due to the broad range of clinical presentations and variable imaging findings. The nonspecific nature of symptoms and lack of systemic features often lead to a delay in diagnosis, which can have catastrophic consequences. Although MRSA is known to cause infections in immunocompromised and hospitalized patients, CA-MRSA infections are becoming increasingly prevalent in the general population, particularly in individuals with predisposing factors such as homelessness and substance use disorder [1,2]. This case highlights the importance of considering MRSA as a potential causative agent in patients presenting with neurological symptoms, especially in vulnerable populations, and emphasizes the need for early recognition and aggressive management of these infections.

## Case presentation

A 45-year-old homeless woman was admitted to the hospital for evaluation of altered mental status. She was brought to the emergency department by emergency medical services (EMS), who found her walking along the streets in a confused state. Her medical history was significant for bipolar disorder, seizure disorder, IV drug use, and substance use disorder. EMS administered Narcan due to suspicion of opiate overdose, but there was minimal response. Upon admission, she exhibited uncooperative and combative behavior. Significant vital signs included a blood pressure of 95/65 mm Hg. The physical examination was unremarkable, except for the neurological exam, which revealed the patient's unresponsiveness to verbal stimuli but the withdrawal of extremities in response to painful stimuli (Glasgow Coma Scale [GCS] was 10), without any focal neurological findings. She was noted to move independently in bed, and there was no evidence of soft tissue or skin infection. 

Initial laboratory workup showed the following results (Table 1) and urinalysis is displayed in Table 2. Urine culture revealed *Escherichia coli* and blood cultures grew MRSA. MRSA screen was negative. The urine toxicology screen was positive for amphetamines. Viral panel including HIV and hepatitis was unremarkable. Thrombophilia workup was negative.

**Table 1:** Initial laboratory workup

Tests	Results	Normal Range
White blood count	17 U/L	4,500-1,000 U/L
Red blood count	5.36 U/L	3.5-5.5 U/L
Hemoglobin	11.7 g/dL	12.0-15.0 g/dL
Hematocrit	39.9%	36.0-47.0%
Neutrophils	15.40 U/L	2.0-7.80 U/L
Lymphocytes	1.30 U/L	1.0-4.8 U/L
Erythrocyte sedimentation rate (ESR)	78 mm/h	0-19 mm/h
C-reactive protein (CRP)	20.16 mg/dL	0-1 mg/dL
Lactic acid	2.6 mmol/L	0.7-2.1 mmol/L

**Table 2:** Urinalysis results WBC: white blood cells; RBC: red blood cells.

	Results	Normal Range
Color	Yellow	Yellow
White blood count	21-50 WBC/hpf	0-5 WBC/hpf
Red blood count	6-10 RBC/hpf	0-2 RBC/hpf
Bacteria	Trace	Negative
Leukocyte esterase	Moderate	Negative
Nitrite	Negative	Negative

**Figure 1:**
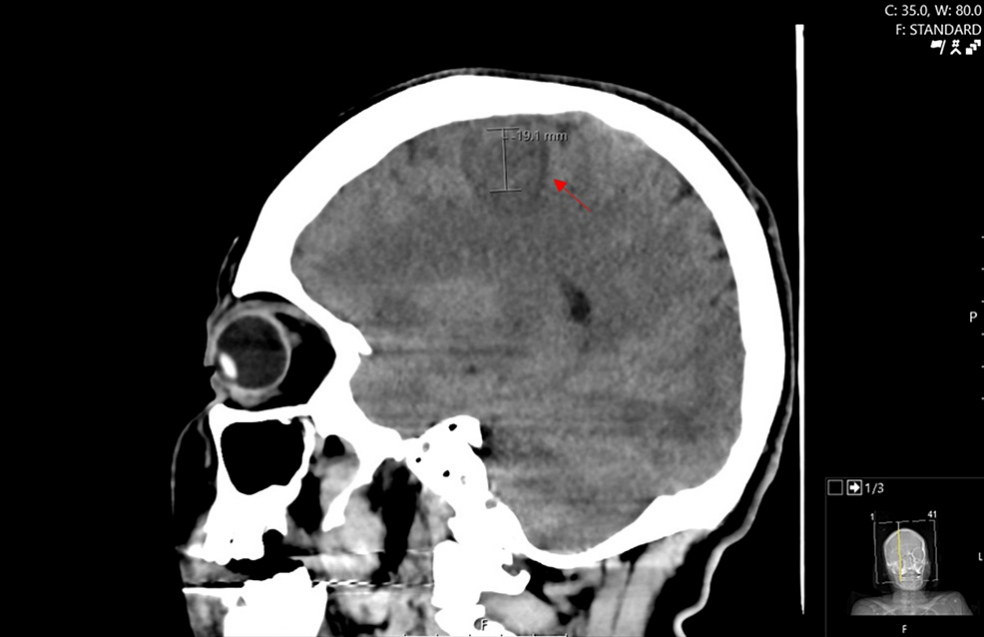
CT scan sagittal plane at admission showing a focal region of hypodensity in the right frontal lobe with surrounding hyperdensity CT: computed tomography.

**Figure 2:**
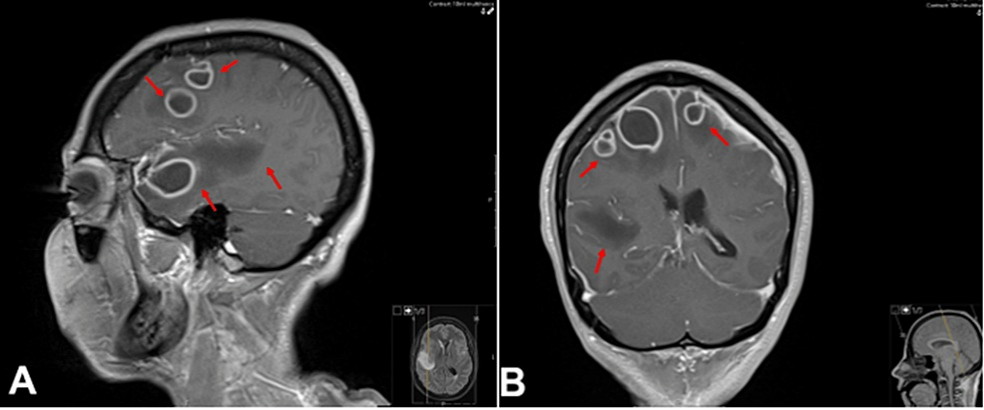
MRI shows multiple right intra-axial lesions with surrounding edema (red arrows) in bilateral frontal lobes and right anterior temporal lobe. (A) Sagittal plane; (B) coronal plane MRI: magnetic resonance imaging.

**Figure 3:**
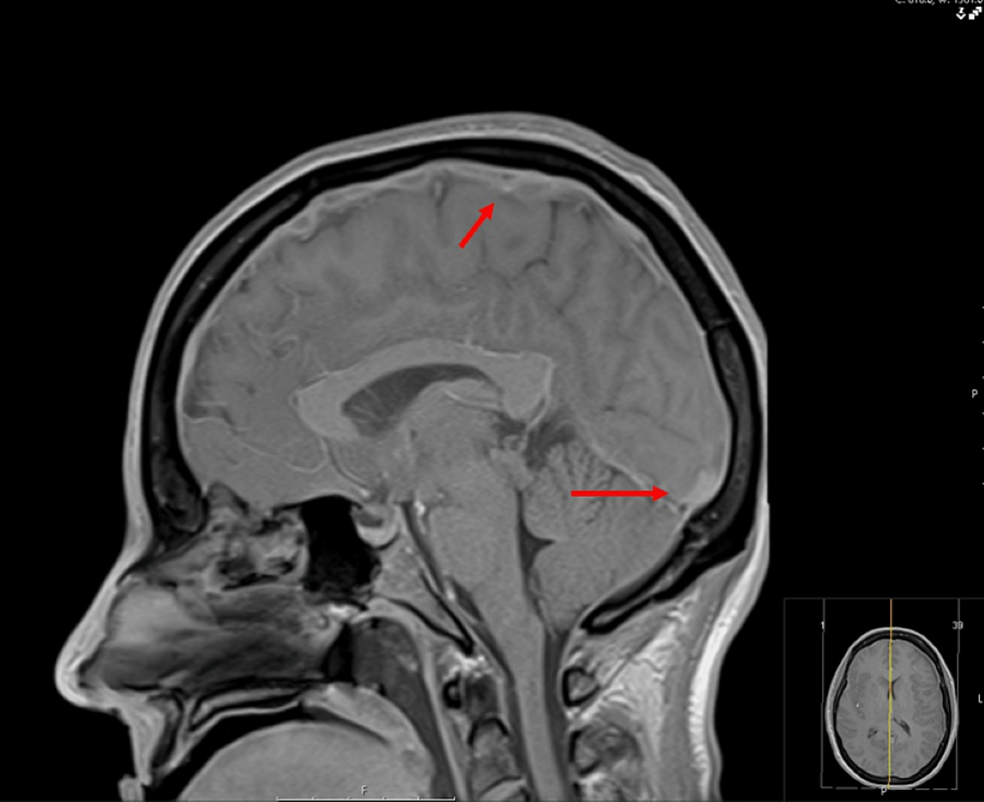
T1-weighted MRI shows sagittal vein thrombosis (sagittal plane) MRI: magnetic resonance imaging.

Initial head CT showed a focal region of hypodensity at the right frontal lobe with surrounding hyperdensity, suspicious for infarct vs mass (Figure 1). MRI brain to further evaluate demonstrated multiple rim-enhancing intra-axial lesions in both hemispheres consistent with cerebral abscesses (Figure 2) as well as sagittal vein thrombosis (Figure 3).

Magnetic resonance angiography (MRA) of the head and neck was unremarkable. Transthoracic echocardiography (TTE) did not show any signs of valvular endocarditis. Additionally, the absence of focal neurological findings at the time of presentation indicated that a lumbar puncture was not necessary.

The clinical presentation along with laboratory and imaging findings in the setting of homelessness, IV drug use, and no recent hospitalization or procedure led to a diagnosis of brain abscesses likely secondary to CA-MRSA. The patient was initially receiving IV vancomycin; however, she was intubated due to worsening mentation and metronidazole, cefepime, and dexamethasone were added to the treatment regimen. Repeat head CT and MRI brain showed the increased size of cerebral abscesses with mass effect. As a result, an emergent right-sided minimally invasive needle biopsy and left frontal craniotomy for abscess evacuation were performed. Cultures of the abscess aspirate grew MRSA, confirming the diagnosis of CA-MRSA brain abscesses. Following the procedure, the patient's clinical status improved, and she was eventually extubated. Additionally, the patient was started on Lovenox (60 mg BID) subcutaneously for sagittal vein thrombosis.

The plan was to continue vancomycin for four weeks, as repeat CT head showed improving edema. However, the patient left against medical advice prior to treatment completion and was lost to follow-up.

## Discussion

This case report highlights a rare presentation of CA-MRSA infection causing multiple cerebral abscesses and sagittal vein thrombosis in a homeless patient with a history of bipolar disorder, seizure disorder, and substance use disorder. Although MRSA infections are becoming increasingly prevalent in the community, cerebral abscesses caused by this pathogen remain rare [3]. *Staphylococcus aureus* is a challenging pathogen in the management and treatment of central nervous system infections, with recent neurosurgical procedures and immunocompromised status as major risk factors, requiring anti-MRSA antibiotics and prompt source control [4]. Furthermore, the presence of sagittal vein thrombosis is an unusual finding in patients with cerebral abscesses, and the combination of these two pathologies poses significant diagnostic and therapeutic challenges [3].

The nonspecific clinical presentation of cerebral abscesses often leads to a delay in diagnosis, which can have catastrophic consequences. The initial presentation and laboratory evidence of the patient in this case report with altered mental status is similar to that seen in other infectious etiologies and non-infectious brain lesions, further complicating the diagnosis. The role of imaging studies, including CT head and MRI brain, in the diagnosis of cerebral abscesses cannot be overstated, as these studies can reveal important diagnostic clues such as the presence of rim-enhancing lesions and surrounding edema [5].

The management of cerebral abscesses caused by MRSA requires a multidisciplinary approach, including the use of IV antibiotics and surgical intervention for abscess evacuation. The treatment of bacterial brain abscess typically involves cefotaxime and metronidazole with additional antibiotics for immunocompromised patients; however, more research is needed to improve understanding of the pharmacokinetic profiles of treatments and the efficacy and safety of drainage and anti-infective treatments in these patients [6]. In this case, the patient underwent a right-sided minimally invasive needle biopsy and left frontal craniotomy for abscess evacuation, which confirmed the diagnosis of MRSA. The early use of broad-spectrum antibiotics, including vancomycin, metronidazole, and cefepime, was crucial in the management of this patient. A retrospective review of adults with pyogenic brain abscesses found that a combined surgical and medical approach with prolonged antimicrobial therapy is necessary to cure the infection and avoid permanent neurologic deficits and that patients managed with medical therapy alone had a higher mortality rate and more neurologic sequelae compared to those who received combined therapy [7]. The lack of recent hospitalization or procedures in the setting of IV drug use and homelessness confirmed that the infection is CA-MRSA. However, the patient left against medical advice before completing treatment, underscoring the challenges in managing vulnerable populations such as the homeless.

This case report highlights the importance of considering CA-MRSA as a potential causative agent in patients presenting with neurological symptoms, particularly in vulnerable populations such as the homeless. The rarity of cerebral abscesses caused by MRSA and the unique presentation of sagittal vein thrombosis in this case further emphasize the diagnostic and therapeutic challenges posed by these infections [2]. This case report serves as a reminder to physicians to maintain a high index of suspicion for CA-MRSA infections in patients presenting with neurological symptoms and to promptly initiate the appropriate treatment to prevent morbidity and mortality.

## Conclusions

This case underscores the diagnostic and therapeutic challenges posed by MRSA infections. Early recognition and prompt treatment of MRSA infections are essential to prevent serious neurological complications such as cerebral abscesses and sagittal vein thrombosis. Multidisciplinary management, including broad-spectrum antibiotics and surgical intervention, is crucial for successful treatment of these infections. Further research is needed to identify the risk factors for MRSA, especially CA-MRSA infections in vulnerable populations, and to develop effective prevention and treatment strategies. Overall, this case report highlights the need for continued vigilance and prompt action in the diagnosis and management of MRSA infections in patients with neurological symptoms.
